# Cone beam CT optimisation for detection of vertical root fracture with metal in the field of view or the exomass

**DOI:** 10.1038/s41598-021-98345-6

**Published:** 2021-09-27

**Authors:** Amanda P. Candemil, Benjamin Salmon, Karla F. Vasconcelos, Anne C. Oenning, Reinhilde Jacobs, Deborah Q. Freitas, Francisco Haiter-Neto, Francesca Mangione, Matheus L. Oliveira

**Affiliations:** 1 Division of Oral Radiology, Department of Oral Diagnosis, Piracicaba Dental School, University of Campinas, Av. Limeira, 901, Piracicaba, São Paulo 13414-903 Brazil; 2 Orofacial Pathologies, Imaging and Biotherapies Lab, Université de Paris, URP2496, Montrouge, France; 3 Dental Medicine Departments, Bretonneau and Henri Mondor University Hospitals, AP-HP, Paris, France; 4 OMFS-IMPATH Research Group, Department of Imaging and Pathology, Faculty of Medicine, Catholic University of Leuven, and Oral & Maxillofacial Surgery, University Hospitals Leuven, Leuven, Belgium; 5 Division of Oral Radiology, Faculdade São Leopoldo Mandic, Instituto de Pesquisas São Leopoldo Mandic, Campinas, Sao Paulo Brazil; 6 Department of Dental Medicine, Karolinska Institute, Stockholm, Sweden

**Keywords:** Cone-beam computed tomography, Endodontics

## Abstract

Dose optimisation has been revisited in the literature due to the frequent use of cone beam computed tomography (CBCT). Although the reduction of the field-of-view (FOV) size has shown to be an effective strategy, this indirectly increases the negative effect from the exomass. The aim of this study was to evaluate the diagnostic accuracy of an optimised CBCT protocol in the detection of simulated vertical root fracture (VRF) in the presence of metal in the exomass and/or inside the FOV. Twenty teeth were endodontically instrumented and VRF was induced in half of them. All teeth were individually placed in a human mandible covered with a soft tissue equivalent material, metallic materials were placed at different dispositions in the exomass and/or endomass, and CBCT scans were obtained at two dose protocols: standard and optimised. Five radiologists evaluated the images and indicated the presence of VRF using a 5-point scale. Area under the ROC curve (AUC), sensitivity, and specificity were calculated and compared using ANOVA (α = 0.05). Overall, AUC, sensitivity, and specificity did not differ significantly (p > 0.05) between the dose protocols. In conclusion, optimised dose protocols should be considered in the detection of simulated VRF irrespective of the occurrence of artefacts from metallic materials in the exomass and/or inside the FOV.

## Introduction

Optimisation is the process of making use of a resource as effectively as possible. When it comes to Radiology, radiation dose optimisation is a protection principle which assures that the X-ray dose delivered to the patient is as low as diagnostically acceptable being indication-oriented and patient specific (ALADAIP)^1–4^. Recently, this concept has been revisited in the scientific community due to the increased application of computed tomography, which presents relatively higher X-ray dose than two-dimensional techniques^5^.

Numerous factors affecting the radiation dose in cone beam computed tomography (CBCT) scan can also influence the final image quality such as the field-of-view (FOV) size, exposure angle, number of collected basis images, exposure time and tube current (milliamperage, mA)^6–8^. Technically, the reduction of these parameters decreases the X-radiation dose; however, the definition of an ideal optimised protocol is challenging because it must also balance the diagnostic task, individual risks of the patient, and inherent aspects of the CBCT unit^4,5^.

The reduction of the FOV size has shown to be an efficient strategy for radiation dose optimisation due to the reduction of the effective dose without compromising the image quality and diagnostic accuracy^9–11^. However, when small FOV sizes are used, all the surrounding structures outside of the FOV but still between the source of X-rays and the image receptor (so-called exomass) have shown to generate image artefacts^12,13^, which can be exacerbated in the presence of highly attenuating materials^13,14^. This is a frequent clinical condition in dentistry because of the wide use of high-density materials in oral rehabilitation, such as titanium implant, ceramic components, gutta-percha, and metallic posts. Interestingly, a recent study^15^ could not observe an effect of metallic materials in exo- and/or endomass on diagnostic accuracy of vertical root fracture (VRF) detection.

CBCT artefacts have shown to decrease the diagnostic accuracy of VRF, which is an undesirable and frequent clinical situation defined as a longitudinally oriented interruption of the dental root from the apex to the coronal portion^16^. The recommended CBCT protocol when VRF is suspected includes a small FOV and, given the microscopic characteristics of this diagnostic task, the highest possible spatial resolution^17–19^; however, the latter is often correlated with higher X-ray dose^20^ due to the need for higher exposure parameters, mainly to increase the contrast-to-noise ratio.

Positive and promising results of optimised CBCT protocols for endodontic purposes are being obtained by using half-scan mode^21–23^ and relatively larger voxel sizes (0.30 mm) at decreased spatial resolution^24^. However, to the best of the authors’ knowledge, the scientific literature has not addressed optimised protocols in the presence of metallic materials in the CBCT exomass for the diagnosis of VRF. Therefore, the aim of this study was to evaluate the diagnostic accuracy of an optimised CBCT protocol for the detection of simulated VRF in the presence of metal artefacts from the exomass and/or endomass.

## Materials and methods

### Ethical aspects

The following methods were carried out in accordance with the Declaration of Helsinki and this study was approved by the Research Ethics Committee of the Piracicaba Dental School of the University of Campinas, Brazil (CAAE: 98690918.9.0000.5418).

### Custom-made exomass phantom

A partially edentulous dry human mandible obtained from the dentomaxillofacial radiology department of Paris University in France was covered with Mix-D, a validated soft tissue simulator of the absorption and scattering of the X-rays^4^. Twenty single-rooted human teeth were extracted for clinical reasons unrelated to the present study and collected after obtaining a written informed consent from all patients, which is in agreement with the Research Ethics Committee of the Piracicaba Dental School of the University of Campinas, Brazil. All teeth had the crown sectioned at the cement-enamel junction by a metallographic cutter (Isomet 1000; Buehler Ltd, Lake Bluff, IL) to avoid bias of memorization of the tooth during the evaluation. The resulting roots were endodontically instrumented (Wave-One primary file system, tip size 25, 0.07 taper, 25 mm, Dentsply Maillefer) using the reciprocating motion (X-Smart Plus, Dentsply Maillefer). VRF was induced in ten teeth, half of the sample, using the international testing machine Instron 4411 (Instron Corporation, Carton, MA) adjusted at 500 N and 1 mm per minute cross-speed. Additionally, to assure the presence of root fracture, all teeth were scanned with the micro-CT unit Quantum FX (PerkinElmer, Waltham, US), adjusted to 160 mA, 90 kVp, 2-min scanning time, FOV size of 20 × 20 mm, and voxel size of 0.04 mm.

**Figure 1:**
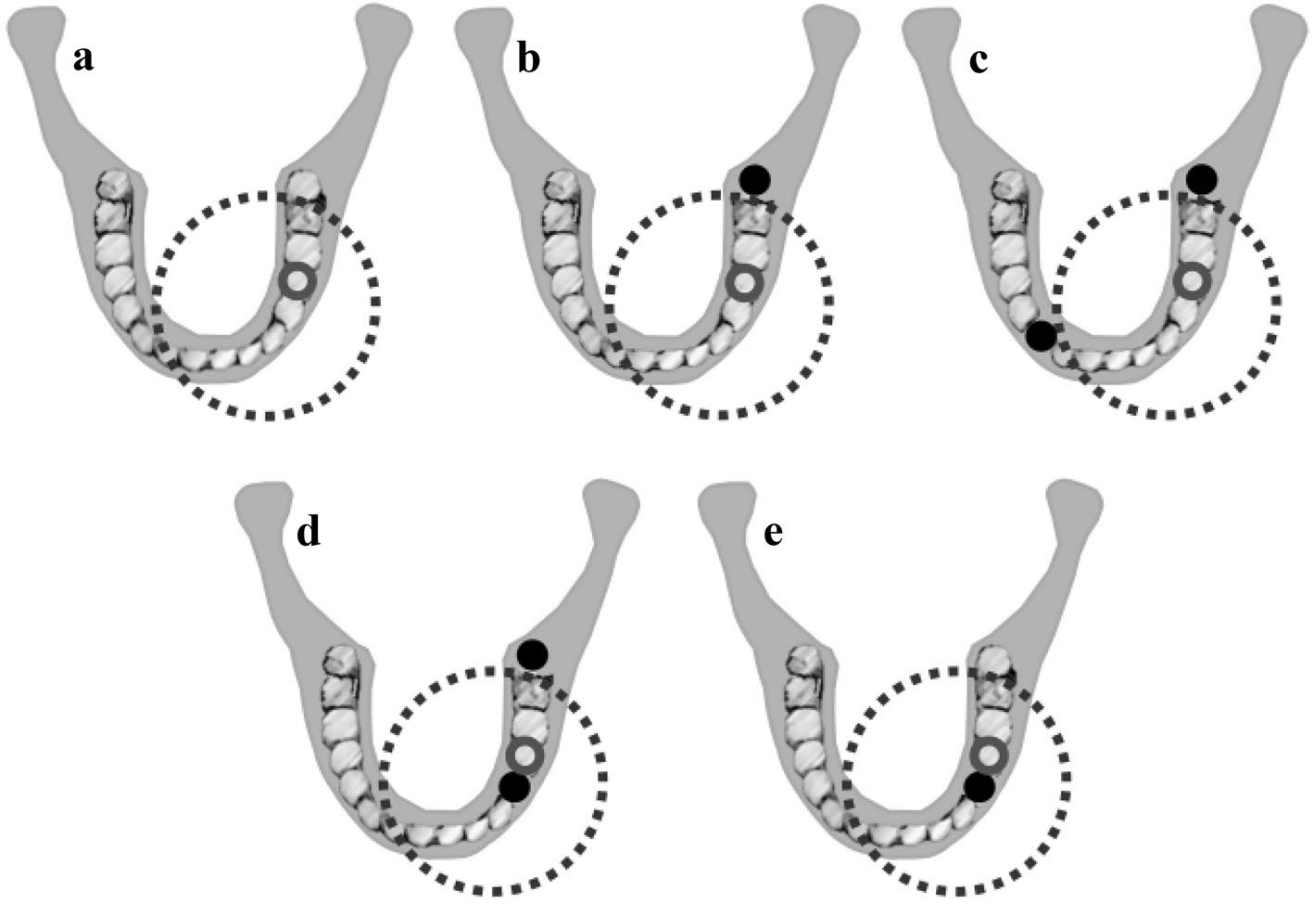
Schematics illustrating the dispositions of the metallic materials in the imaging phantom. The grey circle represents the region of interest (socket of the lower left second premolar), the black dotted circle highlights the limit of the field-of-view and the black solid circle indicates the location of the metallic materials. (**a**) Control (absence of metallic material); (**b**) I Exo—one metallic material in the exomass; (**c**) II Exo—two metallic materials in the exomass; (**d**) ExoEndo—one metallic material in the exomass and one metallic material in the endomass; (**e**) Endo—one metallic material in the endomass.

To simulate a frequent clinical condition without deterioration of the CBCT image arising from the intracanal material of the tooth of interest^25^, a fiberglass post (diameter, 1 mm; height, 10 mm; WhitePost DC, FGM, Joinville, Brazil) composed of fiberglass, epoxy resin, radiopaque compound, inorganic load, and polymerization promoters was inserted into the root canals of all teeth, which were individually placed in the empty socket of the left second premolar of the human mandible. Finally, to simulate a wide range of dispositions of metallic materials in the oral cavity, titanium implants (diameter, 3.5 mm; height, 10 mm; KOPP, Curitiba, PR, Brazil) and cobalt-chromium intracanal posts (cobalt-chromium alloy, Talmax, Curitiba, PR, Brazil) were alternatively placed at four different locations in the exomass and inside of the FOV (endomass), as follows: I Exo—one metallic material in the exomass (in left third molar socket), II Exo—two metallic materials in the exomass (in the right canine and left third molar sockets), ExoEndo—one metallic material in the exomass (in the left third molar socket) and one metallic material in the endomass (in the left first premolar socket), and Endo—one metallic material in the endomass (in the left first premolar socket) (Fig. 1).

### CBCT scans and X-ray dose protocols

**Figure 2:**
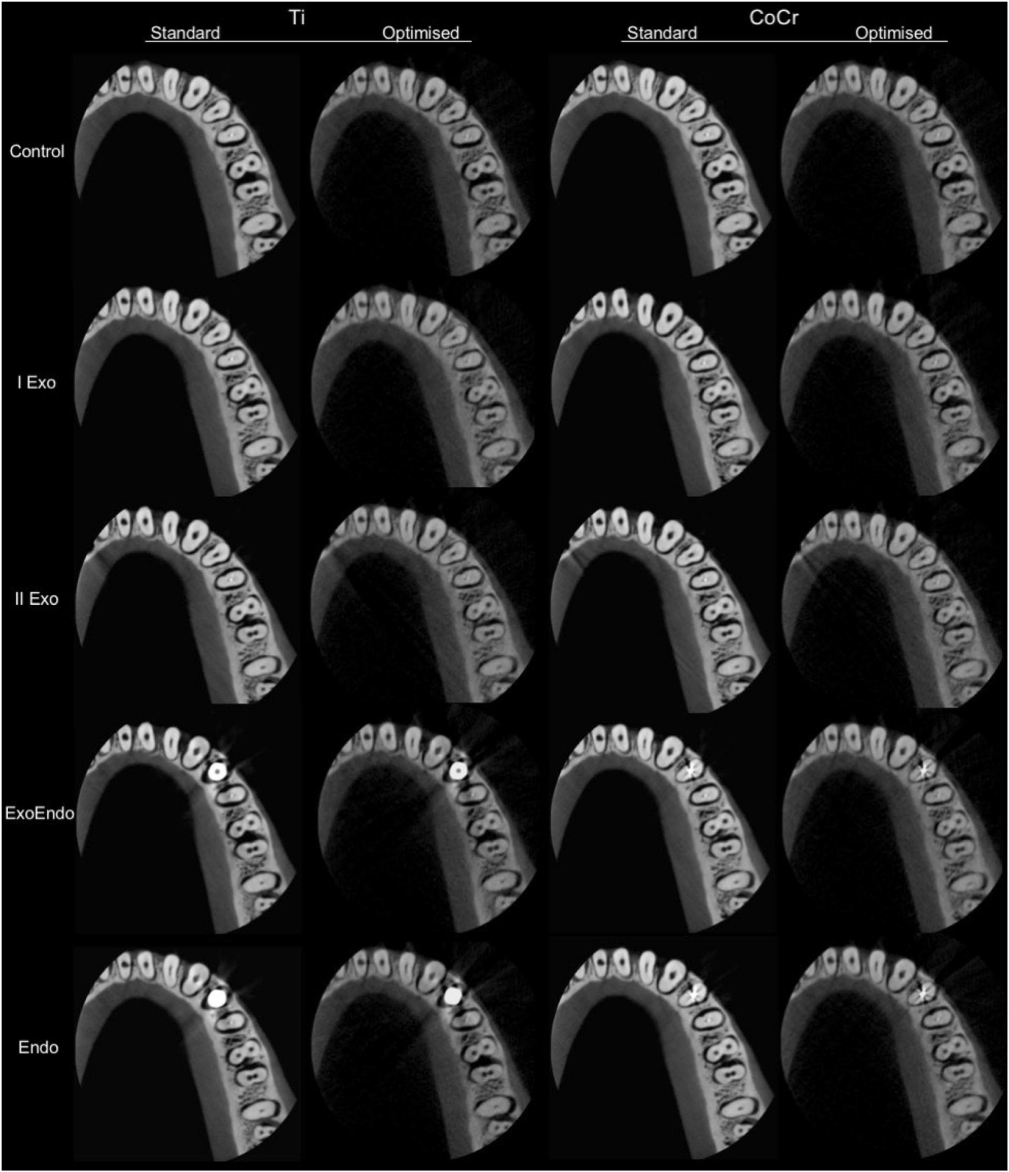
Representative CBCT axial reconstructions of the standard and optimised protocols in different dispositions of titanium implants and cobalt-chromium intracanal posts in the mandible.

**Figure 3:**
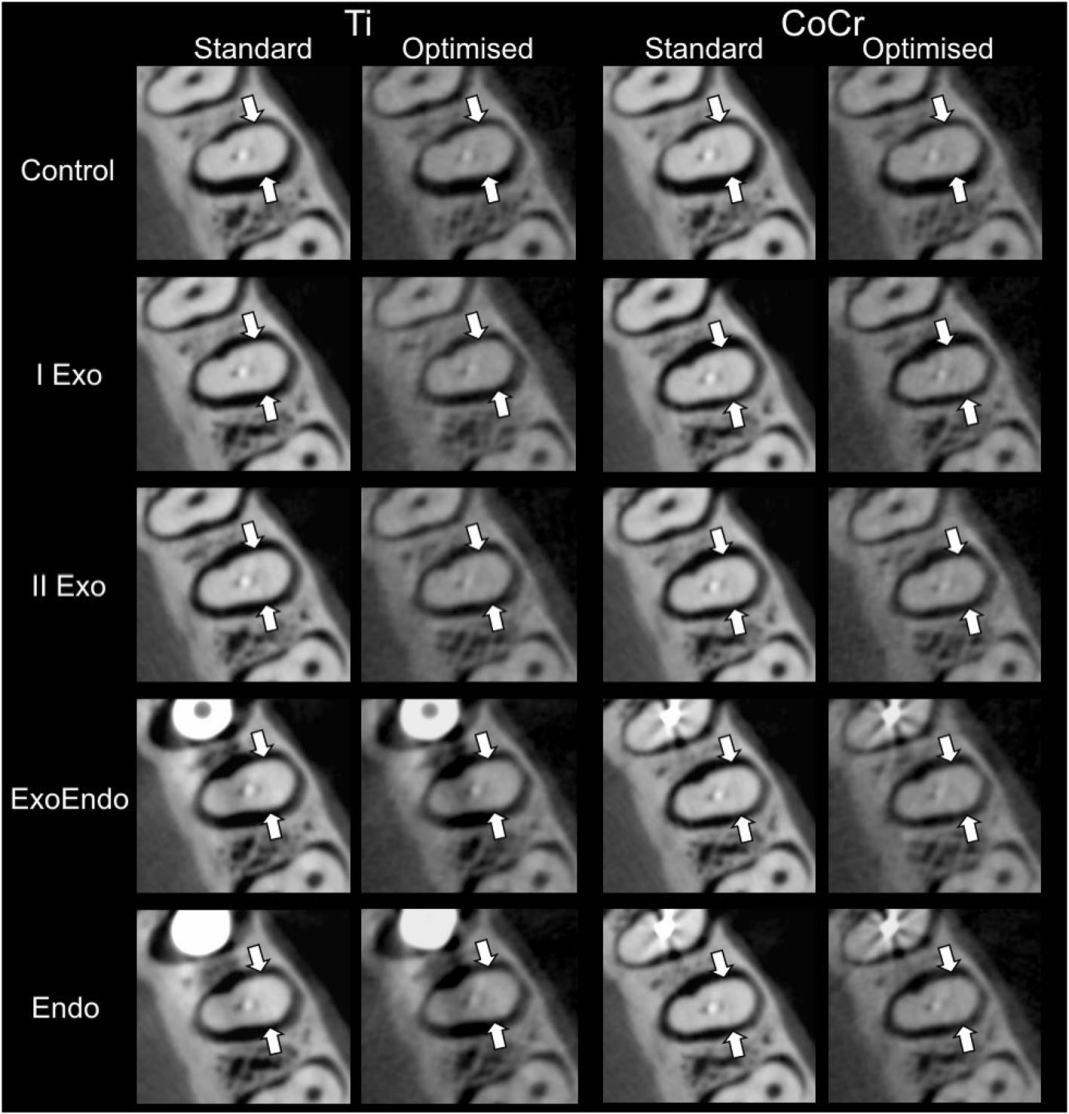
Cropped axial reconstructions of the standard and optimised CBCT protocols in different dispositions of titanium implants and cobalt-chromium intracanal posts in the mandible. The white arrows highlight the vertical root fracture in the second premolar.

For each of the twenty prepared teeth, the imaging phantom was scanned without any metallic material in the exomass (control) and with metallic materials of two compositions alternatively placed at the four dispositions previously described using the CBCT unit CS 9300 (Carestream, Rochester, NY, United States) adjusted to a FOV of 5 × 5 cm, voxel size of 0.09 mm, and two dose protocols: standard (100 mAs, 90 kVp, and a dose-area-product of 7.13 mGycm^2^) and optimised (24 mAs, 70 kVp, and a dose-area-product of 0.86 mGycm^2^) (Figs. 2, 3). The standard protocol was based on the manufacturer’s settings and the optimised protocol was based on the study of Oenning et al.^26^ that showed considerable decrease of the effective dose, calculated by means of a customized Monte Carlo framework, at reduced levels of mA, kVp, and exposure time with an acceptable image quality in the same CBCT unit used in this study.

The resulting volumetric data were exported in DICOM format, imported into OsiriX MD software (Pixmeo Sarl, Geneva, Switzerland) and spatially realigned such that the axial reconstructions were perpendicular to the long axis of the tooth of interest. Furthermore, the window width was adjusted to 5559 and window level to 1779 in all CBCT datasets to avoid bias during image assessment related to the subjective perception of each observer. Those threshold values were previously determined by showing multiple CBCT images adjusted at different window widths and levels to six experienced radiologists who had to vote for the best one. Subsequently, the full stack of axial reconstructions of each CBCT scan was exported and made available to the observers, blinded for the experimental conditions.

### VRF analysis

All the axial stacks were assessed by five oral and maxillofacial radiologists with over 10 years of experience, blinded and previously calibrated, for the detection of VRF. The evaluators were calibrated and trained before the beginning of the assessment. They classified the presence of VRF using a scale of 5 points: 1, absolutely absent; 2, presumably absent; 3, uncertain; 4, presumably present; and 5, absolutely present. The assessment was done in a quiet and darkened room and, to avoid visual fatigue, a limit of 25 volumes per day and an interval of 24 h between sessions were respected.

A revaluation of 25% of the CBCT volumes of each experimental group (with and without fracture, standard and optimised dose protocols and, with and without metallic materials at different dispositions and compositions) were performed after 30 days to analyse intra-observer confidence.

### Statistical analysis

The SPSS software, version 25 (SPSS, Chicago, IL, USA) and GraphPad Prism 8.0 (GraphPad Software, LA Jolla, CA, USA) were used to perform all the analyses with a significance level of 5% (α = 0.05). The area under the receiver operating characteristic curve (AUC), sensitivity, and specificity were calculated for each observer, averaged, and compared among the experimental groups using two-way ANOVA with *post-hoc* Tukey test. In the present study, the AUC was used to measure the diagnostic accuracy of VRF, while sensitivity and specificity assessed the ability of the examiners to correctly identify the presence and absence of VRF. The AUC of each observer was calculated considering the 5 scores and using a public domain web-based calculator for ROC Curves developed at the Johns Hopkins University (Baltimore, Maryland, USA), available at http://www.rad.jhmi.edu/jeng/javarad/roc/JROCFITi.html. To calculate sensitivity and specificity, the scores from the observers were dichotomised such that scores 1 to 3 were classified as absence of VRF and scores 4 and 5 were classified as presence of VRF.

Weighted Kappa test was used to measure the intra- and interobserver agreements and the results were interpreted according to Landis and Koch^27^ (0.00–0.20, poor; 0.21–0.40, reasonable; 0.41–0.60, moderate; 0.61–0.80, good; 0.81–1.00, excellent). A post hoc power analysis was calculated using the software package Bioestat 5.0.^28^.

## Results

In most of the dispositions of the metallic materials, the AUC (standard, 0.89–0.97; optimised, 0.80–0.91), sensitivity (standard, 0.84–0.92 optimised, 0.64–0.78), and specificity (standard, 0.86–0.98; optimised, 0.74–0.94) values did not differ significantly (p > 0.05) between the dose protocols, except when a cobalt-chromium intracanal post or a titanium implant were in the endomass, in which the AUC and sensitivity, respectively, were significantly lower (p < 0.05) for the optimised protocol. When comparing the composition of the metallic materials, no significant difference was found for AUC (p > 0.05), sensitivity (p > 0.05) and specificity (p > 0.05) between titanium and cobalt-chromium (Table 1).

**Table 1:** Mean values (standard deviation) of the area under the receiver operating characteristics curve (AUC), sensitivity, and specificity for different dose protocols, material dispositions and compositions.

	Dose protocol	Control	Material	Dispositions			
				I Exo	II Exo	ExoEndo	Endo
AUC	STD	0.93 (0.09)	Ti	0.97 (0.04)	0.95 (0.05)	0.89 (0.13)	0.93 (0.08)
			CoCr	0.94 (0.09)	0.95 (0.04)	0.92 (0.09)	0.93 (0.06)
	OPT	0.91(0.06)	Ti	0.89 (0.09)	0.91 (0.06)	0.83 (0.05)	0.82 (0,04)
			CoCr	0.82 (0.05)	0.84 (0.10)	0.84 (0.08)	0.80 (0.07)*
Sensitivity	STD	0.84 (0.15)	Ti	0.92 (0.08)	0.88 (0.08)	0.84 (0.21)	0.92 (0.13)
			CoCr	0.88 (0.13)	0.88 (0.08)	0.84 (0.15)	0.86 (0.11)
	OPT	0.78 (0.13)	Ti	0.78 (0.23)	0.76 (0.13)	0.72 (0.13)	0.64 (0.05)*
			CoCr	0.76 (0.18)	0.72 (0.13)	0.70 (0.12)	0.64 (0.11)
Specificity	STD	0.94 (0.09)	Ti	0.98 (0.04)	0.96 (0.05)	0.86 (0.26)	0.88 (0.18)
			CoCr	0.94 (0.09)	0.96 (0.05)	0.94 (0.09)	0.94 (0.09)
	OPT	0.94 (0.09)	Ti	0.84 (0.19)	0.90 (0.14)	0.74 (0.32)	0.94 (0.09)
			CoCr	0.80 (0.12)	0.80 (0.17)	0.92 (0.13)	0.86 (0.09)

Asterisk indicates significantly lower sensitivity than the standard protocol for the same material and disposition.

Both the intraobserver (0.42–0.81) and interobserver (0.53–0.80) agreements ranged from moderate to good. The power analysis revealed that a power of 80% was achieved with 5 observers.

## Discussion

Radiation dose reduction in diagnostic imaging is an appropriate precaution as long as the resulting image presents sufficient quality to be diagnostically acceptable; this is the concept from which the principle of optimisation in radiology is based upon^5,26^. In this respect, the present study was based on an exposure protocol already proven to offer reduced effective dose while maintaining overall image quality, referred to as optimised^26^, and another protocol considered as standard by the manufacturer. As a result, an overall absence of significant differences was found between both dose protocols in the diagnostic accuracy of VRF in the presence of metal artefacts from the exomass and/or endomass.

When comparing the exposure settings of the two dose protocols used in the present study, the optimised protocol made use of reduced mAs (from 100 to 24) and kVp (from 90 to 70), which resulted in an eightfold reduction of the dose-area-product (from 7.13 to 0.86 mGycm^2^). Because the mAs is more efficient for dose reduction and the kV alone may not have a direct and linear influence on the effective dose^29^, it is important to highlight that the optimised protocol of the present study was based on the study by Oenning et al.^26^, which also made use of human phantoms and the same CBCT unit to evaluate six scanning protocols adjusted at varying exposure settings for the visualisation of specific anatomical features; they found that reduced levels of mA, kV, and exposure time resulted in reduced effective dose, calculated by means of a customized Monte Carlo framework. Also, in the present study a smaller FOV size (5 × 5 cm) was used as such to increase the exomass as compared to the previous study (8 × 8 cm)^26^. Attention should be paid to the fact that the relationship between literature-based optimised protocols, effective dose, and image quality, should vary among different CBCT units^6^.

Some exposure settings, such as the mAs and kV, are normally pre-adjusted by the manufacturer that is supposed to have considered the diagnostic task, patient size and age when aiming for better image quality^9^. The mAs has a directly proportional linear relationship with the number of X-ray photons since it affects the number of electrons available in the cathode of the tube when X-rays are produced. Additionally, the kV is responsible for the voltage at which the electrons are subjected and, consequently, for the energy of the resulting X-ray photons, which affects the balance between photoelectric and Compton effects when interacting with the matter^3,11,30^. As previously mentioned, unlike mAs, kV does not have a linear relationship with effective dose^29^. Therefore, kV selection should be carefully optimised to the specific indication for a specific CBCT unit, given that a lower-energy spectrum may be absorbed or scattered within the tissues resulting in higher effective dose. Pauwels et al.^30^ studied the isolated and combined effect of mAs and kV on the radiation dose and contrast-to-noise ratio, and suggested that optimisation in CBCT should be mostly based on mAs reduction because the highest kV value used demonstrated less image degradation even at lower dose levels. Conversely, other studies on dose optimisation showed that both the mAs (from 105 to 52.5 and 157.5 to 87.5) and kV (from 90 to 80) can be reduced without significant impact in the accuracy of diagnostic tasks such as assessment of impacted maxillary canine and periodontal structures^2,31^.

Optimised CBCT protocols should consider several key-points such as patient age, size, and sex, justification criteria and, mostly, the balance between risks and benefits of the examination^26^. The European Society of Endodontology^19^ and the American Association of Endodontists guidelines^17,18^ advise the use of limited FOV CBCT for endodontic purposes; furthermore, the scientific literature has shown positive results of optimised CBCT protocols by using half-scan to detect root fracture^22,23^ and lower mAs and half-scan to detect external root resorption^21,24^. Importantly, unlike from the present study, none of these studies considered the presence of metallic materials in the scanned area, which can cause artefacts on CBCT images and negatively influence the diagnostic accuracy^32^. Bechara et al.^22^ made use of endodontically treated teeth with gutta-percha and showed a significant increase of false-positive diagnosis of root fracture when the number of basis images was halved, due to an increase of beam hardening artefact in the image by gutta-percha. However, the accuracy and sensitivity did not vary significantly.

Because high-density materials are frequently used in oral rehabilitation, the indication of small FOV CBCT for endodontic purposes increases the possibility of localizing them in the exomass. It is therefore important to consider this condition for the study of optimised protocols as high-density materials in the exomass have shown to negatively impact the CBCT image quality^13,14^. Conversely, those materials in the exomass have also shown not to affect the diagnostic accuracy of VRF^15^. In the present study, the presence of titanium implants or cobalt-chromium intracanal posts in the exomass and/or endomass did not influence the diagnosis of VRF at both standard and optimised protocols.

To only analyse the effect of artefacts arising from metallic materials around the tooth of interest, in the exomass and/or endomass, the present methodological design made use of fiberglass endodontic posts in the teeth of interest; they are currently used to reduce the tension of the root in an aesthetic restoration of endodontically treated teeth^33^. Previous studies have shown higher diagnostic accuracy of root fracture and less occurrence of CBCT artefacts in the presence of fiberglass post, when compared with gutta-percha and metallic alloys posts^25,34^. This can be possibly attributed to the different composition of these materials, considering that the higher atomic number of gutta-percha and metallic alloys posts produce more CBCT artefacts, such as hypodense streaks, which mimic fracture lines and increase false-positive diagnosis^35,36^.

The most positive aspects of studying VRF by means of an ex-vivo experimental model include the possibility of having rigorous control of the study variables and rescanning the same condition without breaking radiation protection principles; these aspects would not have been reached with actual patients. Conversely, some inherent limitations cannot be neglected when interpreting our results such as the absence of clinical information, medical history, and possible patient movement during the CBCT scan, which would have reduced image sharpness.

When designing the present study, pilot data and previous studies served the decision to use the full stack of axial reconstructions of all CBCT scans. This decision may have assisted standardize image assessment as axial imaging is preferred to assess VRF, meanwhile avoiding potentially uncontrolled variables from the free use of multiplanar reconstructions, such as observer-dependent oblique assessment, brightness and contrast adjustment. Importantly, previous studies^37,38^ already demonstrated that axial reconstructions are the most accurate CBCT slice orientation for VRF. Also, the relatively high diagnostic values associated with very low values of standard deviation obtained from the five examiners reinforces the absence of possible negative interference from this method on the outcomes of the present study.

Regarding the statistical analysis, none of the observers selected score 3 (uncertain) during image assessment. This is positive for highlighting greater confidence from the observers in the detection of the presence or absence of VRF and for favouring the calculation of the AUC, sensitivity, and specificity.

Overall, the optimised CBCT protocol assessed in this study was applicable without a significant impact on the diagnosis of VRF. Despite the wide number of CBCT units available in the market presenting different configurations for scanning^39^, it is important to highlight that the outcomes of the present study encourage the search for dose optimisation for multiple diagnostic tasks. Further assessment of other CBCT units is needed to establish machine-specific dose-optimised protocols with solid indications and limitations in the diagnosis of VRF.

## Conclusion

Optimised CBCT protocols should be considered in the detection of VRF of dental roots filled with fiberglass posts irrespective of the occurrence of artefacts from metallic materials in the exomass and/or endomass.

## Data Availability

All the data that support the findings of the current study are available from the corresponding author upon reasonable request.
